# Meningococcal carriage in the African meningitis belt

**DOI:** 10.1111/tmi.12125

**Published:** 2013-05-18

**Keywords:** Africa, MenAfriCar, meningococcal carriage, meningococcal vaccines, meningococcus, *Neisseria meningitidis*

## Abstract

A meningococcal serogroup A polysaccharide/tetanus toxoid conjugate vaccine (PsA-TT) (MenAfriVac#x2122;) is being deployed in countries of the African meningitis belt. Experience with other polysaccharide/protein conjugate vaccines has shown that an important part of their success has been their ability to prevent the acquisition of pharyngeal carriage and hence to stop transmission and induce herd immunity. If PsA-TT is to achieve the goal of preventing epidemics, it must be able to prevent the acquisition of pharyngeal carriage as well as invasive meningococcal disease and whether PsA-TT can prevent pharyngeal carriage needs to be determined. To address this issue, a consortium (the African Meningococcal Carriage (MenAfriCar) consortium) was established in 2009 to investigate the pattern of meningococcal carriage in countries of the African meningitis belt prior to and after the introduction of PsA-TT. This article describes how the consortium was established, its objectives and the standardised field and laboratory methods that were used to achieve these objectives. The experience of the MenAfriCar consortium will help in planning future studies on the epidemiology of meningococcal carriage in countries of the African meningitis belt and elsewhere.

Un vaccin conjugué contenant un polysaccharide du sérogroupe A méningococcique et une anatoxine du tétanos (PsA-TT) (MenAfriVac™) est en cours de déploiement dans les pays de la ceinture africaine de la méningite. L’ expérience avec d’ autres vaccins conjugués polysaccharide/protéine a montré qu’ une partie importante de leur succès a été leur capacité à empêcher l’ acquisition du portage pharyngé et donc à arrêter la transmission et à induire une immunité de group. Si PsA-TT doit d’ atteindre l’ objectif de prévenir les épidémies, il devrait être en mesure d’ empêcher l’ acquisition du portage pharyngé ainsi que la méningococcie invasive et le fait que PsA-TT puisse empêcher le portage pharyngé devrait être déterminé. Pour résoudre ce problème, le consortium MenAfriCar (Consortium Africain du Portage Méningococcique) a été établi en 2009 pour étudier le mode de portage du méningocoque dans les pays de la ceinture africaine de la méningite avant et après l’ introduction de PsA-TT. Cet article décrit comment le consortium a été établi, ses objectifs et les méthodes de laboratoire et de terrain standardisées qui ont été utilisées pour atteindre ces objectifs. L’ expérience du consortium MenAfriCar aidera à planifier les futures études sur l’ épidémiologie du portage du méningocoque dans les pays de la ceinture africaine de la méningite et d’ ailleurs.

Se está utilizando una vacuna meningocócica conjugada (MenAfriVac™) de polisacárido del serogrupo A / tétano toxoide (PsA-TT) en países del cinturón Africano de meningitis. Las experiencias obtenidas con otras vacunas conjugadas polisacárido/proteína han demostrado que una parte importante de su éxito se debe a su habilidad para prevenir la colonización faríngea de los portadores, acabando por lo tanto con la transmisión, y a la de inducir la protección de rebaño. Si PsA-TT ha de cumplir el objetivo de prevenir epidemias, debe ser capaz de prevenir el estado de portador faríngeo, al igual que la enfermedad invasiva por meningococo, y para ello es necesario determinar si la PsA-TT puede prevenir la colonización faríngea. Con el fin de abordar esta cuestión se estableció un consorcio africano en el 2009 - el MenAfriCar (*African Meningococcal Carriage Consortium*) – para investigar los patrones del estado de portador de meningococo en países del cinturón Africano de la meningitis, antes y después de la introducción de PsA-TT. Este artículo describe como se estableció el consorcio, sus objetivos y los métodos estandarizados de campo y de laboratorio que se utilizaron para alcanzarlos. La experiencia del consorcio MenAfriCar ayudará en la planificación de estudios futuros sobre la epidemiología del estado de portador de meningococo, tanto en países del cinturón Africano de la meningitis como en otros lugares del mundo.

## Introduction

For at least 100 years, epidemics of meningococcal meningitis have occurred frequently but unpredictably in a region of the Sahel and sub-Sahel known as the African meningitis belt (Lapeyssonie 1963). Most African epidemics have been caused by meningococci belonging to serogroup A (Greenwood 1999) but outbreaks caused by meningococci belonging to serogroup C, W or X meningococci have been reported (Broome *et al*. 1983; Decosas & Koama 2002; Boisier *et al*. 2007; Halperin *et al*. 2012).

For the past three decades, control of epidemic meningococcal disease in the African meningitis belt has relied upon reactive vaccination with a polysaccharide vaccine once the incidence of cases in a district or region has passed a critical incidence threshold (WHO Working Group 1998). When reactive vaccination with polysaccharide vaccines has been deployed early in an epidemic, it has saved many lives but it has not reduced the frequency of epidemics because polysaccharide vaccines induce protection of limited duration, especially in children, and have little or no impact on carriage (Dellicour & Greenwood 2007). Polysaccharide/protein conjugate vaccines are likely to be more successful in preventing epidemics because they induce immunological memory and decrease pharyngeal carriage.

Widespread deployment of serogroup C meningococcal conjugate vaccines in Europe has reduced serogroup C meningococcal disease and carriage and led to elimination of the serogroup C meningococcal clone responsible for outbreaks in the UK (Maiden & Stuart 2002; Trotter *et al*. 2004). Unfortunately, Africa has had to wait many years longer than the industrialised world for an affordable meningococcal conjugate vaccine that could prevent serogroup A epidemics. However, following an innovative and highly successful development programme undertaken by the Meningitis Vaccine Project (MVP), a collaboration between WHO and PATH, a monovalent serogroup A conjugate (PsA-TT) (MenAfriVac^™)^), manufactured by the Serum Institute of India, was licensed in India in 2009 and pre-qualified by WHO in 2010 (Frasch *et al*. 2012). At the end of that year, mass vaccination programmes in subjects aged 1–29 years were launched in Burkina Faso, Mali and Niger (Djingarey *et al*. 2012). Recent reports suggest that the vaccine has been highly effective in reducing serogroup A meningococcal disease and carriage in Burkina Faso (Kristiansen *et al*. 2012; Novak *et al*. 2012).

PsA-TT, like other conjugate vaccines, was pre-qualified on the basis of its safety and immunogenicity (Sow *et al*. 2011) without an efficacy trial being undertaken. Therefore, post-implementation evaluation of its impact on both meningococcal disease and carriage is essential. Consequently, in 2009, the African Meningococcal Carriage (MenAfriCar) consortium was established to measure the prevalence of meningococcal carriage across the African meningitis belt using standardised techniques before and after introduction of PsA-TT. This article describes how the consortium was established, its objectives and the methods used to meet these objectives.

## Creation of the African meningococcal carriage consortium

Several years before a serogroup A meningococcal conjugate vaccine became a reality, a group of scientists interested in meningococcal disease in Africa began discussions on the importance of measuring the impact of such a vaccine on carriage and the challenges that this would pose. Few meningococcal carriage studies have been conducted in the African meningitis belt in recent years (Leimkugel *et al*. 2007; Mueller *et al*. 2007; Kristiansen *et al*. 2011), and earlier studies gave very varied results because they had been restricted to a single site, had been conducted at different times of the year and/or used a variety of methods (Trotter & Greenwood 2007). Therefore, it was agreed that the best way forward would be the formation of a group that could carry out carriage studies across the meningitis belt before and after introduction of the new vaccine using standardised methods. Consequently, the African Meningococcal Carriage (MenAfriCar) consortium, comprising seven African and 12 northern partners (Box 1), was established formally in 2009.

**Box 1. Members of the African Meningococcal Carriage consortium**

**African Partners:** Armauer Hansen Research Institute, Addis Ababa, Ethiopia; Centre de Recherche Médical et Sanitaire (CERMES), Niamey, Niger; Centre pour les Vaccins en Développement (CVD), Bamako, Mali; Institut de Recherche pour le Développement (IRD), Dakar, Senegal; Centre de Support en Santé Internationale (CSSI), N’Djamena, Chad; Navrongo Health Research Centre, Navrongo, Ghana; University of Maiduguri (Department of Community Medicine), Maiduguri, Nigeria. **Northern Partners:** Agence de Médecine Préventive (AMP), Paris, France; Centers for Disease Control (Division of Bacterial Diseases), Atlanta, USA; Health Protection Agency (Vaccine Evaluation Unit), Manchester, UK; Institut Pasteur (Unité Infections Bactériennes Invasives), Paris, France; Institut de Médecine Tropical du Service de Santé des Armées (IMTSSA), Marseille, France; London School of Hygiene & Tropical Medicine, London, UK; Norwegian Public Health Institute, Oslo, Norway; PATH (Meningitis Vaccine Project), Ferney, France; Swiss Tropical and Public Health Institute, Basle, Switzerland; University of Bristol (Department of Social Medicine), Bristol, UK; University of Oxford (Department of Zoology), Oxford, UK; World Health Organisation, Geneva, Switzerland.

## Objectives of the consortium

The objectives of the MenAfriCar consortium are summarised in Box 2.

**Box 2. Objectives of the African Meningococcal Carriage consortium**

Determination of the prevalence of meningococcal carriage in seven countries extending across the meningitis belt from east to west (Ethiopia, Chad, Nigeria, Niger, Ghana, Mali and Senegal) prior to the introduction of PsA-TT.

Characterisation of risk factors for carriage of meningococci in countries of the African meningitis belt.

Investigation of the spread of meningococci within households.

Measurement of the impact of PsA-TT on serogroup A meningococcal carriage.

Measurement of serogroup A meningococcal antibody concentrations by ELISA and bactericidal assays in countries across the meningitis belt.

## Organisation and management of the consortium

Each centre identified a principal investigator whose team has been supported by two epidemiologists and a data manager based at the London School of Hygiene & Tropical Medicine (LSHTM) and by a laboratory manager based at the Centre for Vaccine Development (CVD), Bamako, Mali. A small secretariat was established at LSHTM to manage the project’s finances, order supplies, organise meetings and coordinate communications. Clinical trial monitors have visited each centre on at least three occasions during the course of carriage surveys, and an international advisory committee has provided overall guidance on the conduct of the project.

Communications officers, assisted by a small committee, were established at the beginning of the project at each African centre and, after formal training, given responsibility for informing study communities about the nature and purpose of the carriage studies, informing these communities of their findings and for communicating results to officials of the Ministry of Health, WHO and other interested parties. A consortium newsletter was developed and circulated widely. An open access website (http://www.menafricar.org) was established as well as a separate, password protected site, accessible only to consortium members, which holds all operational documents and which hosts a discussion forum. Each centre established a crisis management committee to manage any unforeseen events, such as a death occurring during the course of a carriage study.

The study was approved by the LSHTM Ethics Committee and by the ethics committees of each of the African partner institutions with the exception of Chad, which does not have a formal ethical committee, where approval was granted by a committee set-up to oversee MenAfriCar studies by the Ministry of Health. The study was registered with http://ClinicalTrials.gov (NCT01119482).

## Study design, populations and epidemiological methods

### Introduction

**Figure 1:**
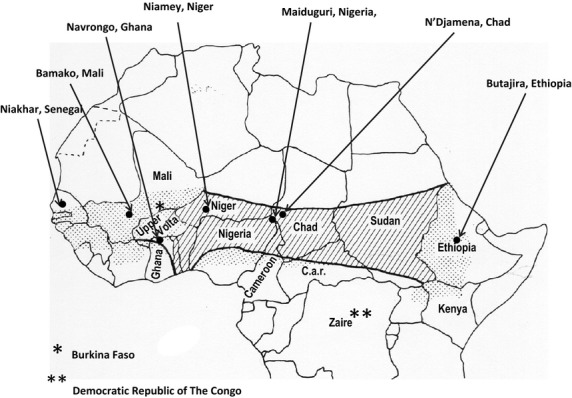
Map of the meningitis belt showing the situation of the study centres sites based on the original description by Lapeyssonnie [1] (hatched areas) and additional areas (dotted) where the epidemiology of meningococcal disease has subsequently been shown to be characteristic of the meningitis belt.

**Figure 2:**
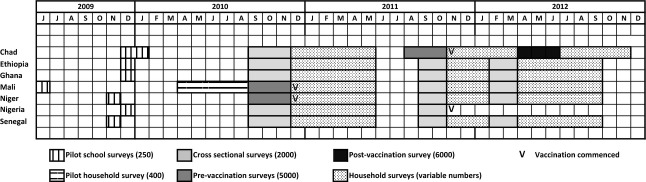
Gantt chart showing the timing of different MenAfriCar carriage surveys.

Carriage studies were conducted in seven countries within the African meningitis belt: Chad, Ethiopia, Ghana, Mali, Niger, Nigeria and Senegal (Figure 1). Both urban and rural study sites were selected in each country. The timing of carriage surveys is shown in Figure 2.

#### Pilot studies

Pilot studies were undertaken at each centre in 2009 or 2010 in a convenience sample of 250 schoolchildren to familiarise the investigators with the standard operating procedures and to determine the optimum method of collecting pharyngeal swabs for the isolation of meningococci. It was found that collecting swabs touching both the posterior pharynx and the tonsils was more effective at isolating *Neisseria meningitidis* than swabbing just the posterior of the pharynx (Basta 2011); thus, this technique was used in all subsequent surveys.

A further pilot study of the methods to be used in household studies was conducted in 2010 in which a sample of 400 individuals resident in over 100 households in Bamako, Mali, were screened for carriage and 200 residents of 20 households followed prospectively for 6 months.

## Pre-vaccination cross-sectional surveys

Three cross-sectional surveys, two in the rainy and one in the dry season, were undertaken between July 2010 and July 2012 (Figure2) in each country except for Nigeria, where the last survey could not be carried out because of political instability. A representative sample frame was obtained either from an updated, ongoing demographic surveillance system (DSS) or from a census conducted specifically for the study. Multistage sampling was used. Study households were selected by simple random sampling from the DSS or census. Within selected households, individuals stratified by age group (<1 year, 1–4 years, 5–14 years, 15–29 years and 30 years or more) were randomly selected until the required sample size had been reached.

In Mali and Niger, the first survey included 5 000 subjects. In Chad, the second and third surveys included at least 5 000 subjects. All other pre-vaccination surveys included approximately 2 000 subjects. The only exclusion criteria for these surveys were lack of consent or being too ill to participate. Once consent and assent were obtained, a structured questionnaire^1^ was completed by the head of the household to obtain information on potential risk factors for carriage at the household level. Each study participant or their guardian completed a short questionnaire, which collected demographic information and information on potential risk factors for meningococcal carriage at the individual level. A pharyngeal swab was obtained and plated out directly in the field. A 5 ml blood sample was obtained from 400 randomly selected, age-stratified subjects aged 6 months or more in each study area during the first cross-sectional survey.

The rate of recruitment during cross-sectional surveys was determined largely by the degree of dispersal of the study subjects and by the ability of laboratory staff to handle the microbiological plates that they received. This worked out at a maximum of 100 samples a day for an individual laboratory with an average of around 50 samples a day.

### Household contact surveys

Longitudinal household surveys were triggered by the identification of an index carrier during a cross-sectional survey. Within 4 weeks of the identification of a carrier, the household of these subjects was visited and all household members were invited to participate in a follow-up study. Once a household had consented to join the study, pharyngeal swabs were collected from household members twice a month for 2 months and then monthly for a further 4 months. During the first visit, the head of the household was asked to complete a detailed household questionnaire. Household members were asked to provide similar information to that requested from volunteers in the cross-sectional surveys. Height and weight of each household contact over 6 months of age who agreed to join the study were measured, and a pharyngeal swab and a 5 ml blood sample were obtained.

### Post-vaccination surveys

It was planned initially to undertake post-vaccination surveys in Mali and Niger, early PsA-TT adopter countries, but as no serogroup A meningococci were detected in pre-vaccination surveys in either of these countries, this plan was not pursued. Instead, a post-vaccination survey in approximately 6 000 individuals was conducted in 2012 in Chad, where serogroup A meningococci had been isolated during the cross-sectional survey conducted prior to vaccination of a part of the country at the end of 2011.

### Sample size

Determination of sample sizes for these carriage studies was difficult because of the variability in carriage prevalence reported in previous African surveys (Trotter & Greenwood 2007). For the pre- and post-vaccination comparison, a sample size of 5 000 was chosen on the grounds of expected carriage rates and logistic constraints. With a baseline serogroup A carriage prevalence of 1% (a figure assumed to be conservative), a study of this size would have 80% power to show a 50% reduction in carriage following vaccination. In the centres where the introduction of PsA-TT was not imminent, the objectives of the carriage surveys were largely descriptive, but a sample size of 2 000 was deemed large enough to enable meaningful comparisons to be made by country, site (urban *vs*. rural), season and age group. Based on previous experience with cross-sectional serological surveys, it was considered that an age-stratified sample of 800 per centre would allow meaningful comparisons of serogroup-specific IgG and serum bactericidal antibody concentrations by age and across countries.

There are no data on the rate of meningococcal acquisition in household contacts in Africa to assist in the design of the household studies. An informed presumption was that the household acquisition rate would be around 1% per month, but some pragmatic decisions were taken regarding the number of households that the field teams had the capacity to recruit and follow.

## Laboratory methods

### Isolation and characterisation of meningococci

Pharyngeal swabs were plated directly onto modified Thayer–Martin agar plates in the field and taken to the laboratory within 6 h of collection where they were incubated for 24–48 h at 37 °C in 5% CO_2_. During a pilot study, one or two colonies on the Thayer–Martin plate with a morphology typical of *Neisseria* species were selected for Gram staining and oxidase testing (Beckton Dickinson, Oxford, UK). Oxidase-positive, Gram-negative colonies were then subcultured onto one blood agar plate containing 7% defibrinated sheep’s blood and these plates incubated for 18–24 h at 37 °C in 5% CO_2_ (Martin *et al*. 1974). Bacteria from the blood agar plate were serogrouped by slide agglutination with meningococcal serogroup A, W and X antisera (Difco, Becton Dickinson, Oxford, UK). A fine suspension of the test organism was made in 10 μl of phosphate-buffered saline (PBS) on a clean glass slide, and 10 μl of antiserum was added to the bacterial suspension. The slide was rocked for 4 min before the agglutination pattern was read.

For the main studies, one suspected colony with a morphology typical of a *Neisseria* species was subcultured onto two blood agar plates and the plates incubated for 18–24 h at 37 °C. Bacteria from one blood agar plate were tested for oxidase activity and then Gram stained. Oxidase-positive, Gram-negative diplococci were then tested for *γ*-glutamyl-transferase activity (GGT) (Rosco Diagnostica, Taastrup, Denmark) (Takahashi *et al*. 2004), *β*-galactosidase activity with ortho-nitrophenyl-*β*-d-galactopyranoside (ONPG) (Rosco Diagnostica,Taastrup, Denmark) for identification of *N. lactamica* and butyrate esterase activity (Tributyrin) (Rosco Diagnostica, Taastrup, Denmark) for distinguishing between *N. meningitidis* and *Moraxella* species (Pérez *et al*. 1990). To carry out these tests, a loopful of an 18- to 24-h culture of the test organism grown on blood agar was suspended in 250 μl of normal saline and the suspension incubated with the appropriate enzyme substrate at 37 °C for 4 h before observation for the indicative colour change. Isolates with the profile GGT positive, ONPG negative and tributyrin negative were characterised as presumptive *N. meningitidis* and serogrouped by slide agglutination using serogroup A, W, X and Y antisera (Difco, Becton Dickinson, Oxford, UK). A fine suspension of the test organism was made directly in each of the four antisera on a clean glass slide and the slide rocked for 1 min. Each antiserum was tested with a positive and negative control bacterium. A sample was assigned to a specific serogroup only if visible agglutination or clumping of the bacteria, with clearing of the background bacterial suspension on the slide, was observed within 1 min (World Health Organization 2009). Bacterial colonies from the second agar plate were used for DNA preparation by boiling the bacteria from half a plate suspended in PBS for 20 min in a water bath. The rest of the bacteria on the same agar plate were prepared for long-term storage on beads (Microbank, Pro Lab Diagnostics, Bromborough, UK), following the manufacturers’ instructions, and the sample stored at −80 °C.

To test the ability of the participating laboratories to correctly identify *Neisseria* growing on Thayer–Martin agar plates, fifty samples from the selective medium from which no *Neisseria* were identified as well as fifty samples from organisms identified as oxidase-positive, Gram-negative diplococci at each site were sent to the WHO Coordinating Centre at the Institut Pasteur, Paris, for confirmation of the results obtained at the African centres. A description of the quality control procedures adopted for this study will be presented in a subsequent paper.

### Molecular characterisation of *Neisseria*

Boiled suspensions of Gram-negative diplococci were separated into four aliquots. One aliquot was sent to the University of Oxford for molecular analysis, the second used on site for identification of *N. meningitidis* by PCR and the remaining two aliquots archived.

Following a workshop at CERMES on PCR detection and genogrouping of meningococci and regular follow-up visits by the laboratory coordinator, six of the seven MenAfriCar centres are now able to conduct a *por A PCR* test to confirm the identity of isolates of *N. meningitidis* identified by routine microbiology. If the *por A* PCR test is positive, an A, X, W multiplex genogrouping PCR is performed. If this first multiplex PCR is negative, a singleplex Y genogrouping PCR is performed. Ready-to-load master mix containing Hot Start Taq polymerase (Solis Byodine, Tartu, Estonia) was chosen as the PCR mixture to render the PCR technique as simple as possible and because this enzyme-containing master mix can be transported at room temperature. The 7.5 mm MgCl_2_ master mix was adjusted to 11 mm MgCl_2_ to optimise the technique. Prior to the introduction of PCR testing as a routine procedure, each site underwent cross-validation of the technique, comparing results of approximately 50 samples with results obtained at the University of Oxford.

On arrival in Oxford, heat-killed bacterial cell suspensions are archived at −20 °C and entered on a BIGSdb database (Jolley & Maiden 2010) for subsequent hierarchical analysis using a custom-built high-throughput Sanger sequencing pipeline. All sequence data are submitted directly into this database and the results interpreted automatically by querying with reference sequences. The first stage of characterisation of *Neisseria* species is done with an assay based on ribosomal multilocus sequence typing (rMLST) (Bennett *et al*. 2012; Jolley *et al*. 2012), which indexes variation in a 413-bp fragment of a gene encoding a ribosomal protein subunit (*rplF*) (Bennett JS, Watkins ER, Jolley KA, Harrison OB, Maiden MC, unpublished). The *rplF* gene fragment is amplified and sequenced. A null result (no sequence) is consistent with the specimen not containing a member of the genus *Neisseria*; sequences obtained from positive specimens are diagnostic for each *Neisseria* species. Isolates that are *rplF* negative are characterised by sequencing a fragment of the 16S (small subunit) rRNA gene to confirm genus or lack of bacterial DNA suitable for PCR amplification. All samples are also tested with an assay that sequences the capsule null (*cnl*) region of the meningococcal chromosome (Claus *et al*. 2002). This region is present in genetically characterised meningococci without a capsule and in all *Neisseria* species other than meningococci. Samples that are identified as meningococci with the *rplF* assay, but which are negative for *cnl*, are characterised for genogroup by amplification and sequencing of a fragment of the *ctrA* gene, which is present in all meningococci with a capsule synthesis (*cps*) region. Sequences of these fragments identify most genogroups (A, E, I, K, L, X, Z), with the organisms with *cps* regions encoding sialic acid-containing capsules (B, C, W, Y) identified by further sequencing the *cssD* gene in their *cps* region (Harrison *et al*. 2013).

**Figure 3:**
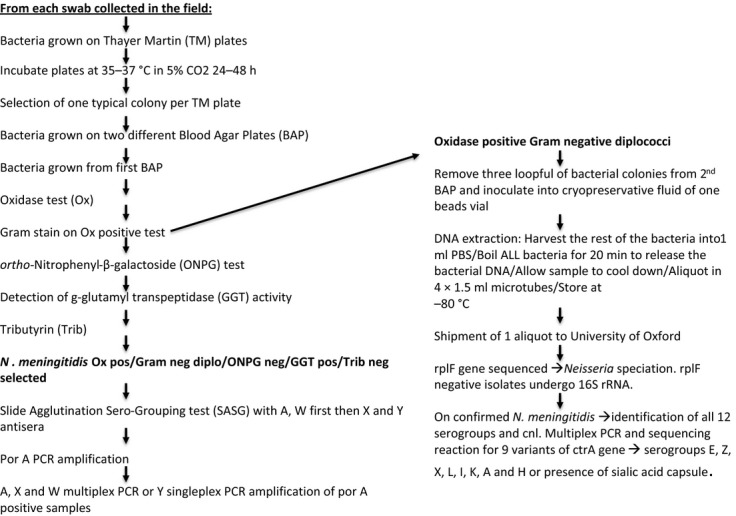
Flow diagram indicating the procedure for isolating and characterising *Nesisseria meningitidis* isolates from oropharyngeal swabs.

The approach taken to the isolation and characterisation of *N. meningitidis* from pharyngeal swabs is summarised in Figure 3.

### Serology

Two serogroup A meningococcal antibody assays have been used in the MenAfriCar studies, a quantitative enzyme-linked immunosorbent assay (ELISA) (Carlone *et al*. 1992) and a serum bactericidal antibody assay that uses rabbit complement (rSBA) Maslanka *et al*. 1997).

To facilitate transfer of the ELISA technique from the Health Protection Agency (HPA) Vaccine Evaluation Unit (VEU), Manchester, UK to Africa, two workshops were organised, one at the HPA VEU predominantly for scientists from the English-speaking countries and the other at CVD, Mali, for those from French-speaking countries. The ELISA has been implemented successfully at six of the seven MenAfriCar centres.

The ELISA used for the MenAfriCar studies is a classical indirect assay involving serial dilutions with each test serum assayed in duplicate. Although simple to perform, interpretation of the results of the assay requires sound mathematical and statistical knowledge. Standard serum CDC 1992, with an assigned serogroup A-specific IgG concentration of 91.8 μg/ml, was used as the reference serum (Holder *et al*. 1995). In addition, a locally calibrated positive control serum sample was included on each plate. The lower limit of quantitation for this ELISA is 0.19 μg/ml. Consistency in the results obtained following testing of the control samples on at least 40 occasions was required before progression to cross-validation.

Before analysis of samples collected during the cross-sectional surveys commenced, the results on 50 samples tested on site and at the HPA VEU were cross-validated. A correlation coefficient of >0.9 was required before a centre progressed to testing of survey samples (Lin 1989). Some centres needed to undergo cross-validation on more than one occasion before meeting this target. Regular monitoring visits and constant support from the MenAfriCar laboratory team were needed to achieve this important step.

As the rSBA is a more complex technique than ELISA and requires expensive equipment, it has been undertaken at only two of the MenAfriCar centres (CVD, Mali and CERMES, Niger), one of which (CERMES) already had experience with this technique. SBA assays were performed using the CDC F8238 strain of serogroup A meningococcus (phenotype A: 4,21: P1.20,9, L11), as described previously (Maslanka *et al*. 1997). The complement source was serum from 3- to 4-week-old rabbits (Pel Freez Biologicals, Wisconsin). Titres were expressed as the reciprocal serum dilution yielding 50% killing after 60-min incubation. Cross-validation of the rSBA with results obtained at the HPA VEU was undertaken on 50 samples. Agreement for routine testing to proceed required that the results of testing on site and at the VEU, Manchester, to be within a minus one, plus one range in titre. Some of the serum samples used for cross-validation had to be excluded from the panel because of inconsistent results obtained after performing runs in triplicate within each laboratory. This is thought to have been due to the presence of low avidity antibodies that may have been naturally induced. Standardisation of the SBA technique proved difficult, and Mali had to repeat the cross-validation exercise twice.

### Data management

Various systems for data management that required construction of local and a central database were considered, including manual double entry, the open-source electronic data capture system Open Clinica (Paulsen *et al*. 2012) and TeleForm, an automated form processing system for data management (TeleForm software: version 10.4.1® 1991-2009, Autonomy). After preliminary tests at several African centres, was TeleForm chosen. This data management system allows study forms to be specially designed, scanned after completion, automatically read through a combination of optical/intelligent character recognition, optical mark recognition and barcode recognition and then verified before being exported to a local database. It also allows rapid data entry and reduces opportunities for error, especially for optical mark recognition variables such as check boxes. For harmonisation purposes and to facilitate technical support, centres were provided with similar scanning and computing equipment pre-loaded with TeleForm.

To ensure that all centres used the same study forms, were developed centrally in both English and TeleForm French. A label form was designed, which could be populated automatically with study IDs and printed in self-adhesive label sheets for use in the laboratory. Local and central databases were designed to accommodate the study forms. A separate database module was developed centrally to automatically populate the main study database with genetic laboratory results from the Oxford BIGS database (Jolley & Maiden 2010). Additional data validation software packages were developed and shared between centres to harmonise local data cleaning. Training sessions on the new system were organised for data managers and field workers, and continuous technical support was provided to them throughout the study.

### Capacity development

An important component of the activities of the MenAfriCar consortium has been strengthening the research capacity of the participating African centres. Since 2010, 13 African scientists supported by the MenAfriCar consortium have received formal training in epidemiology, public health, microbiology, data or project management. Four scientists have been supported to undertake a MSc course, three have attended a short course on genomics and clinical microbiology at the Sanger Centre, Cambridge, UK, and two spent several months at the University of Oxford gaining practical experience in molecular microbiological techniques. Training in data management for scientists from Ethiopia has been provided at LSHTM and at the KEMRI-Wellcome Laboratories, Kilifi, Kenya.

In addition to the PCR and serology training workshops mentioned above, a one-week course on good clinical and laboratory practice (GCP and GLP) was organised by LSHTM and the Contract Laboratory Services (CLS) in Johannesburg, South Africa. Formal training for the study teams in microbiology and serology has been supplemented by regular visits to each centre by the senior scientists participating in the project.

## Discussion

The study described in this article has been the first large, multicountry meningococcal carriage study conducted in the African meningitis belt using a standardised study protocol. Many lessons were learnt during the course of the project, which involved both francophone and anglophone countries, which could be of value in the design of future carriage studies.

The unpredictability of meningococcal infection in the African meningitis belt makes forward planning of epidemiological studies difficult. Therefore, the consortium initiated carriage studies in seven countries across the meningitis belt in the expectation that the serogroup A meningococcus would be circulating in at least one of these countries during the period of the study, as proved to be the case. This flexibility allowed the consortium to adapt the design of its studies as additional information about the prevalence, and serogroup of carriers was obtained. Specifically, post-vaccination activities were transferred from Mali and Niger to Chad when it emerged that with the exception of northern Cameroon, this was the only country in the African meningitis belt with substantial transmission of serogroup A meningococci in 2011 and 2012.

Carriage of *N. meningitidis* is relatively uncommon in the general population between epidemics, necessitating large samples to demonstrate differences in carriage rates between populations or over time. On the basis of the limited information available, we estimated that the overall carriage rate of *N. meningitidis* would be around 10% and that of serogroup A, meningococci would be about 1% outside epidemic periods. These estimates were over optimistic, and a serogroup A carriage rate approaching 1% was found only in rural areas of Chad. It is likely that the MenAfriCar carriage studies have been undertaken at a time of a naturally low level of serogroup A meningococcal transmission in the African meningitis belt, but experience from these studies indicates that future carriage studies to monitor the impact of conjugate vaccines in the African meningitis belt are likely to require many thousands of subjects to ensure sufficient power to detect significant differences between groups.

All the participating African centres had previous experience of field studies, and no major problems were encountered in the implementation of the carriage surveys. Selection of households for inclusion in the carriage studies was relatively straightforward at the sites where a demographic surveillance system was in place but required a preliminary census in those that did not. Practical problems were encountered when households selected for inclusion in a survey were widely scattered, requiring the study team to repeatedly pack up and move the equipment needed for collection of swabs and blood samples to another site, slowing down the daily rate of recruitment. Local solutions to this problem were developed, which included bringing individuals selected for inclusion in the survey to a common meeting point (Ghana) and deployment of up to 16 field teams to work concurrently (Chad). The rate at which subjects could be recruited was limited by the rate at which a single laboratory could handle the samples collected. This proved to be about 100 samples a day. As expected, the speed and efficiency of carriage surveys improved progressively during the course of the study.

To ensure that results obtained at each of the seven centres in the consortium could be compared, it was essential that reproducible and standardised laboratory methods were used at each centre. Ensuring that, this was the case proved more difficult than had been anticipated originally. Quality control of both microbiological and serological assays was facilitated through support from the northern partners in the consortium. Quality control in microbiological techniques was helped greatly by the molecular characterisation of isolates at the Centre de Recherche Médicale et Sanitaire (CERMES), Niamey, the Norwegian Institute of Public Health (NIPH), Oslo and at the University of Oxford. This provided early indication of problems at some centres with both speciation and serogrouping of neisserial isolates. This allowed remedial actions, including introduction of biochemical testing after the pilot studies and modification of the serogrouping agglutination assay followed by training workshops after the first cross-sectional survey, to be undertaken early in the project. An additional wet season survey was undertaken, a dry season survey postponed for 12 months and quality control procedures reviewed in the light of early experiences. The importance of a detailed quality control procedure for studies of the kind undertaken by the MenAfriCar consortium has recently been stressed by Kristiansen *et al*. (2012) based on their experience in Burkina Faso. These investigators adopted a similar approach to that employed in the MenAfriCar project, utilising both conventional and molecular techniques but Kristiansen *et al*. were more rigorous in their application of conventional microbiological techniques by repeating these on meningococci sent to the NIPH.

The ELISA technique used for measuring IgG antibodies to meningococcal polysaccharides has been developed to the standard required for registration of new meningococcal vaccines and transferring this technology to Africa proved challenging. It is possible that a simpler technique would be adequate for epidemiological studies. However, this assay, and the SBA assay, are being used to measure immunological correlates of protection against serogroup A meningococcal carriage in household and longitudinal studies and it is therefore important that the quality of these assays meets high internationals standards.

A primary objective of the MenAfriCar consortium has been the collection of comparable data across the centres. The TeleForm data management adopted for the study was new to most centres and because of lack of time for training and frequent changes in form design at the start of the project, completion errors and poor optical character recognition were initially common. However, this situation improved as form design became stable and field workers and local data managers gained experience and confidence in completing forms and using the system. Slow and intermittent Internet connectivity was initially a major challenge that led to delays in uploading local data and synchronising local and central databases, which, in turn, affected central monitoring. This challenge was partially addressed by the end of the project with some centres acquiring higher bandwidth or dedicated Internet connection, for example, through mobile broadband, and by others performing data upload operations in the evening or at night. By the end of the study, all centres were able to upload on schedule.

Initial results from evaluation of the impact of PsA-TT in Burkina Faso are encouraging with marked drops in the incidence of serogroup A meningitis and carriage following introduction of the vaccine (Kristiansen *et al*. 2012; Novak *et al*. 2012). However, there will be a continuing need to evaluate the impact of this and other meningococcal vaccines on meningococcal carriage as well as invasive disease in countries of the African meningitis belt during the coming years. Experience gained by members of the MenAfriCar consortium will help in meeting this challenge.

## Note

Copies of risk factor questionnaires are available from the authors.

## Appendix 1

Armauer Hansen Research Institute, Addis Ababa, Ethiopia: Oumer Ali, Abraham Aseffa (PI), Ahmed Bedru, Tsehaynesh Lemma, Tesfaye Moti, Alemayehu Worku, Haimanot Guebre Xabher, Lawrence Yamuah.

Centre de Recherche Médicale et Sanitaire (CERMES), Niamey, Niger (Member of the International Network of Pasteur Institutes): Rahamatou Moustapha Boukary, Jean-Marc Collard (PI), Ibrahim Dan Dano, Ibrahim Habiboulaye, Bassira Issaka, Jean-François Jusot, Sani Ousmane, Issoufa Rabe.

Centers for Disease Control, Atlanta, USA: Tom Clark, Leonard Mayer.

Centre de Support en Santé International (CSSI), N’Djamena, Chad: Jean Pierre Gami, Kadidja Gamougam, Boulotigam Kodbesse, Nathan Naibei, Cyriaque Ngadoua, Lodoum Mbainadji, Daugla Doumagoum Moto (PI), Maxime Narbé, Jacques Toralta.

Centre pour les Vaccins en Développement, Bamako, Mali: Abdoulaye Berthe, Mahamadou Keita, Kanny Diallo, Uma Onwuchekwa, Samba O Sow (PI), Boubou Tamboura, Awa Traore, Alou Toure.

Department of Community Medicine, University of Maiduguri, Maiduguri, Nigeria: Mary Amodu, Omeiza Beida, Galadima Gadzama, Babatunji Omotara (PI), Zailani Sambo, Shuaibu Yahya.

Faculty of Infectious Disease, London School of Hygiene & Tropical Medicine, London, UK: Daniel Chandramohan, Brian Greenwood, Musa Hassan-King, Olivier Manigart, Maria Nascimento, James Stuart, Arouna Woukeu.

Health Protection Agency Vaccine Evaluation Unit, Manchester, UK: Xilian Bai, Ray Borrow, Helen Findlow.

Institut de Recherche pour le Développement, Dakar, Senegal: Serge Avalo, Hubert Bassene, Aldiouma Diallo (PI), Marietou Dieng, Souleymane Doucouré, Jules François Gomis, Assane Ndiaye, Cheikh Sokhna, Jean François Trape.

Navrongo Health Research Centre, Navrongo, Ghana: Bugri Akalifa, Abudulai Forgor, Abrahan Hodgson (PI), Isaac Osei, Stephen Quaye, John Williams, Peter Wontuo.

Princeton University USA: Nicole Basta (Formerly University of Washington)(supported by NIH grants 2R37A1032042 and R03A1092121).

School of Social and Community Medicine, University of Bristol, Bristol, UK: Thomas Irving; Caroline Trotter.

University of Oxford, UK: Julia Bennett, Marietou Dieng, Dorothea Hill, Odile Harrison, Lisa Rebbetts, Martin Maiden, Yenenesh Tekletsion, Eleanor Watkins.

## References

[b1] Basta N (2011). University of Washington. The epidemiology of Neisseria meningitidis carriage in Bamako, Mali prior to the introduction of a newly developed meningococcal serogroup A vaccine. Thesis for the Degree of Doctor of Philosophy,.

[b2] Bennett JS, Jolley KA, Earle SG (2012). A genomic approach to bacterial taxonomy: an examination and proposed reclassification of species within the genus *Neisseria*. Microbiology.

[b3] Boisier P, Nicolas P, Djibo S (2007). Meningococcal meningitis: unprecedented incidence of serogroup X-related cases in 2006 in Niger. Clinical Infectious Diseases.

[b4] Broome CV, Rugh MA, Yada AA (1983). Epidemic group C meningococcal meningitis in Upper Volta 1979. Bulletin of the World Health Organization.

[b5] Carlone GM, Frasch CE, Siber GR (1992). Multicenter comparison of levels of antibody to the *Neisseria meningitidis* group A capsular polysaccharide measured by using an enzyme-linked immunosorbent assay. Journal of Clinical Microbiology.

[b6] Claus H, Maiden MC, Maag R (2002). Many carried meningococci lack the genes required for capsule synthesis and transport. Microbiology.

[b7] Decosas J, Koama JB (2002). Chronicle of an outbreak foretold: meningococcal meningitis W135 in Burkina Faso. Lancet Infectious Diseases.

[b8] Dellicour S, Greenwood B (2007). Impact of meningococcal vaccination on pharyngeal carriage of meningococci. Tropical Medical and International Health.

[b9] Djingarey MH, Barry R, Bonkoungou M (2012). Effectively introducing a new meningococcal A conjugate vaccine in Africa: The Burkina Faso experience. Vaccine.

[b10] Frasch C, Preziosi MP, Laforce FM (2012). Development of a group A meningococcal conjugate vaccine, MenAfriVac™. Human Vaccine Immunotherapy.

[b11] Greenwood BM (1999). Meningococcal meningitis in Africa. Transactions of the Royal Society of Tropical Medicine and Hygiene.

[b12] Halperin SA, Bettinger JA, Greenwood B (2012). The changing and dynamic epidemiology of meningococcal disease. Vaccine.

[b100] Harrison OB, Claus H, Jian Y (2013). Description and nomenclature of Neisseria meningitidis capsule locus. Emerging Infectious Diseases.

[b13] Holder PK, Maslanka SE, Pais LB (1995). Assignment of *Neisseria meningitidis* group A and C class specific anticapsular antibody concentrations to the new standard reference serum CDC1992. Clinical Diagnostic Laboratory Immunology.

[b14] Jolley KA, Maiden MC (2010). BIGSdb: Scalable analysis of bacterial genome variation at the population level. BMC Bioinformatics.

[b15] Jolley KA, Bliss CM, Bennett JS (2012). Ribosomal multi-locus sequence typing: universal characterization of bacteria from domain to strain. Microbiology.

[b16] Kristiansen PA, Diomandé F, Wei SC (2011). Baseline meningococcal carriage in Burkina Faso before the introduction of a meningococcal serogroup A conjugate vaccine. Clinical Vaccine Immunology.

[b17] Kristiansen PA, Ouédraogo AS, Sanou I (2012). Laboratory quality control in a multicentre meningococcal carriage study in Burkina Faso. Transactions of the Royal Society of Tropical Medicine and Hygiene.

[b18] Kristiansen PA, Diomandé F, Ba AK (2013). Impact of the serogroup A meningococcal conjugate vaccine, MenAfriVac, on carriage and herd immunity. Clinical Infectious Diseases.

[b19] Lapeyssonie L (1963). La méningite cérébrospinale en Afrique. Bulletin of the World Health Organization.

[b20] Leimkugel J, Hodgson A, Forgor AA (2007). Clonal waves of *Neisseria* colonisation and disease in the African meningitis belt: eight-year longitudinal study in northern Ghana. PloS Medicine.

[b21] Lin LI (1989). A concordance correlation coefficient to evaluate reproducibility. Biometrics.

[b22] Maiden MCJ, Stuart JM, UK Meningococcal Carriage Group (2002). Carriage of serogroup C meningococci 1 year after meningococcal C conjugate polysaccharide vaccination. Lancet.

[b23] Martin JE, Armstrong JH, Smith PB (1974). New system for cultivation *of Neisseria gonorrhoeae*. Applied Microbiology.

[b24] Maslanka SE, Gheesling LL, Libutti DE (1997). Standardization and a multilaboratory comparison of *Neisseria meningitidis* group A and C serum bactericidal assays. The Multilaboratory Study Group. Clinical Diagnostic Laboratory Immunology.

[b25] Mueller JE, Sangaré L, Njanpop-Lafourcade B-M (2007). Molecular characteristics and epidemiology of meningococcal carriage, Burkina Faso 2003. Emerging Infectious Diseases.

[b26] Novak RT, Kambou JL, Diomandé FV (2012). Serogroup A meningococcal conjugate vaccination in Burkina Faso: analysis of national surveillance data. Lancet Infectious Diseases.

[b27] Paulsen A, Overgaard S, Lauritsen JM (2012). Quality of data entry using single entry, double entry and automated forms processing - an example based on a study of patient-reported outcomes. PLoS ONE.

[b28] Pérez JL, Pulido A, Pantozzi F (1990). Butyrate esterase (4-methylumberllifery butyrate) spot test, a simple method for immediate identification of *Moraxella (Branhamella) catarrhalis*. Journal of Clinical Microbiology.

[b29] Sow SO, Okoko BJ, Diallo A (2011). Immunogenicity and safety of a meningococcal A conjugate vaccine in Africans. New England Journal of Medicine.

[b30] Takahashi H, Kuroki T, Watanabe Y (2004). Reliability of the detection of meningococcal gamma-glutamyl transpeptidase as an identification marker for *Neisseria meningitidis*. Microbiological Immunology.

[b31] Trotter CL, Greenwood BM (2007). Meningococcal carriage in the African meningitis belt. Lancet Infectious Diseases.

[b32] Trotter CL, Andrews NJ, Kaczmarski EB (2004). Effectiveness of meningococcal serogroup C conjugate vaccine 4 years after introduction. Lancet.

[b33] WHO Working Group (1998). Control of epidemic meningococcal disease: WHO practical guidelines.

[b34] World Health Organization (2009). Laboratory methods for the diagnosis of meningitis caused by Neisseria meningitidis, Streptococcus pneumoniae, and Haemophilus influenzae.

